# Initial step-up treatment changes in asthmatic children already prescribed inhaled corticosteroids: a historical cohort study

**DOI:** 10.1038/npjpcrm.2015.41

**Published:** 2015-06-11

**Authors:** Steve W Turner, Kathryn Richardson, Annie Burden, Mike Thomas, Clare Murray, David Price

**Affiliations:** 1 Child Health, University of Aberdeen, Aberdeen, UK; 2 Research in Real Life, Cambridge, UK; 3 Primary Care, University of Southampton, Southampton, UK; 4 Manchester Academic Health Science Centre, The University of Manchester, Manchester, UK

## Abstract

**Background:**

When standard doses of inhaled corticosteroids (ICS) fail to control symptoms in children aged >4 years, guidelines recommend the addition of a long-acting β_2_-agonist (LABA), with other treatment options being available if symptoms persist.

**Aims:**

To determine the proportion of initial ‘step-up’ episodes where LABAs were prescribed and to describe characteristics of individuals not stepped up with LABA.

**Methods:**

Between 1999 and 2011, initial step-up episodes from ICS monotherapy were identified in children aged 5–12 years with asthma and in receipt of ICS. Data sources were the Clinical Practice Research Datalink and Optimum Patient Care Research Database.

**Results:**

Initial step-up episodes were identified in 10,793 children. ICS dose was increased in 6,252 children (58%), LABA was introduced in 3,436 (32%; including 1,107 where fixed dose combination inhaler (FDC) replaced the ICS inhaler), and leukotriene receptor antagonist (LTRA) was added in 1,105 (10%). Compared with children stepped up to any LABA, others were younger and prescribed lower doses of ICS and reliever medication. ICS dose increase was more likely in obese children and LTRA prescribing was more likely in children with rhinitis and in receipt of antibiotics. Compared with FDC, step-up to separate LABA inhaler was more likely in younger, obese children who were using less oral steroids.

**Conclusions:**

One-third of initial step-up episodes in children with asthma treated with ICS are to add LABA. Different characteristics of children prescribed therapies other than LABA suggest that prescribers tailor treatment in some clinical settings.

## Introduction

Asthma is a common condition^1^ and 5% of children in the UK are prescribed inhaled corticosteroids (ICS) to control asthma symptoms.^2^ Annually there are over 25,000 children admitted to hospital for asthma in England and Wales^3^ and the cost of asthma inhaler prescriptions issued in the UK to children aged 5–15 years is ~£37million,^4^ which is equivalent to £5 for every child in the population. Treatment with low dose ICS is effective in controlling symptoms for the majority of children but ~10% of children require one of four additional ‘step-up’ treatment options.^1,2^ Clinical trials have demonstrated that many children will benefit from more than one step-up option^5–10^ but equally, some will gain greatest benefit from one of the options.^5,10^

In the absence of evidence for a ‘best’ step-up option, guidelines for asthma management in children advocate either addition of LABA^1,11^ or increasing ICS dose^12^ as the first step-up option while highlighting the need to assess response and prescribe an alternative treatment for non-responders. More recent guidance recommends that step-up to fixed dose combination inhaler (FDC) containing ICS and LABA is preferable to addition of separate LABA inhaler due to better adherence to a single inhaler device.^13^ What is not known is whether these guidelines^1,13^ have been put into practice and our main research question was ‘in what proportion of first step-up episodes is LABA treatment added to ICS rather than ICS dose increased or LTRA added?’. Given the uncertainty as to which initial treatment step-up is most effective, we anticipated that some children would be stepped up to treatment other than LABA and tested the null hypothesis that children stepped up to LTRA or increased ICS would be no different to those stepped up to LABA; the presence of differences between those stepped up to LABA and to other medications would suggest that prescribers recognise subgroups who they feel are more likely to respond to options other than LABA. We tested our research question and hypothesis using routinely acquired ‘real life’ data from primary care, an approach which complements results from clinical trials^14^ and has been used in the paediatric setting^15^ and also, in adults, in the context of stepping up treatment.^16^

## Materials and methods

### Study population

Prescribing data were obtained from the CPRD and the OPCRD between 1 January 1990 and 23 December 2011 for OPCRD and 11 April 2011 for CPRD. Data before 1 January 1999 were not included since LTRA and FDC inhalers were not licensed for use in the UK until 1998 and 1999, respectively. Inclusion criteria were (i) asthma diagnosis, defined as a Read code for asthma and/or >1 prescription for asthma medications (including ⩾1 for ICS) in the 12 months prior to the first step-up date (index date), (ii) aged 5–12 years when treatment stepped up to allow comparison with recommendations for this age range set out in the BTS/SIGN guideline,^1^ (iii) registered with the practice for at least 12 months prior to and post index date. Exclusion criteria were (i) other chronic respiratory disease, (ii) maintenance oral steroid prescribed in the 12 months prior to the index date, (iii) previous prescription for LABA (as separate or FDC inhaler), LTRA or theophylline in the year prior to the index date, (iv) multiple step-up therapies, multiple ICS, or prescription of both FDC and ICS inhalers at index date, (v) ICS step-up dose by <50%,^17^ and (vii) prescribed theophylline at step-up (due to small numbers).

### Study design

Patients were categorized according to their first step-up as: (i) addition of LABA, ICS dose unchanged, (ii) increase ICS dose, (iii) addition of LTRA, ICS dose unchanged, and (iv) replacement of ICS inhaler with FDC. Distinction was made between FDC and addition of separate LABA inhaler since the former is the recommended option.^13^ This study was approved in 2010 by the Independent Scientific Advisory Committee of the (then) General Practice Research Database.

### Predictor variables (or patient characteristics)

Patient characteristics and asthma medication use were defined over the 12 months prior to step-up. Rhinitis diagnosis was ‘rhinitis ever’ as coded in primary care (Read) records. Eczema medications were those prescribed in the 12 months prior to step-up. SABA use was defined as any prescription in the baseline year. Body mass index (BMI) was derived from height and weight and converted to centiles and *z*-scores with reference to the 1990 UK standard^18^ applying clinical cutoffs for overweight (⩾91st centile) and obese (⩾98th centile). BMI *z*-scores outside the range ±5 were excluded as presumed data entry mistake.^19^ Paracetamol prescription in the previous year was included since this is a confounder in adult studies.^20^ Ethnicity data were not available. The online supplement describes the methods for deriving the following variables: average daily ICS dosage, average daily short-acting β_2_-agonist (SABA) use, acute oral steroid use, and medication possession ratio.

### Clinical practice research datalink

The Clinical practice research datalink (CPRD), initially the General Practice Research Database, was established in 1990 and by 1999 included a stable population of 650 practices across the UK and provided primary care prescribing for ~5% of the population. (http://www.cprd.com/intro.asp).

### Optimum patient care research database

The Optimum Patient Care Research Database (OPCRD) was established in 2008 and currently has 424 practices in the database. Patients with respiratory conditions are actively recruited. The OPCRD holds data from 1,200,014 patients including 514,719 with asthma; therefore OPCRD has data on ~10% of the UK asthma population. Algorithms identify any patients included from both CPRD and OPCRD and remove duplicates. (http://www.optimumpatientcare.org/Html_Docs/OPCRD.html).

### Statistical analysis

Differences across the categories of ICS dose increases and also the univariate associations between first post ICS step-up therapy and each patient characteristic or baseline asthma medication variable were tested using a *Χ* ^2^-test for categorical variables and Kruskal–Wallis for variables measured on the interval or ratio scale. A multivariate regression model was built using multinomial logistic regression with FDC as the reference group, by first including age, sex, index year, and all variables with univariate associations with *P*<0.1 in the model. Variables were examined for co-linearity and clinical importance then removed in a backwards stepwise procedure until all variables remaining in the multivariate model had *P*<0.1. Since addition of LABA has until recently been the recommended first step-up option, we repeated analyses with this option as the reference. All analyses were carried out using SAS version 9.3 (SAS, Cary, NC, USA).

## Results

### Study subjects

**Figure 1:**
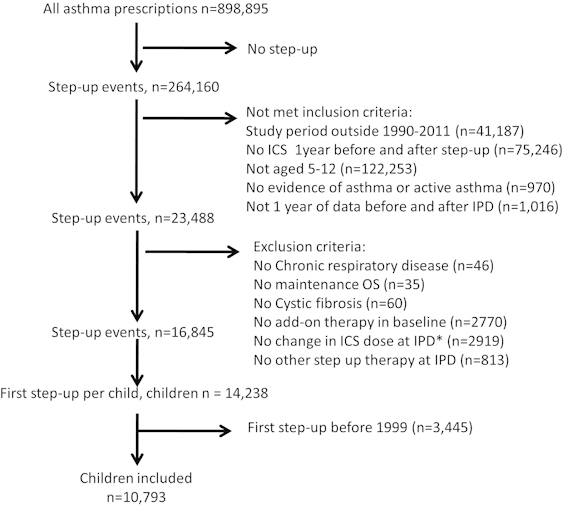
CONSORT diagram showing how the individuals included in the present study were identified within the CPRD and OPCRD databases. *IPD=index prescription date.

Data were available in 10,793 individuals (7,609 from OPCRD) whose median (interquartile range) age was 9 (6–11) years, 59% were male, 97% had a diagnosis of asthma, and 50% were prescribed eczema medications (Table 1). Figure 1 shows how individuals were identified from the databases. There were 6,252 children where ICS dose was increased (58%), 2,329 were prescribed a separate LABA inhaler (22%), 1,107 switched to FDC (10%), and 1,105 had LTRA added (10%). Table 2 describes the characteristics of individuals where ICS dose was increased stratified by dose increase option. These seven groups included 96% of all ICS dose step-up episodes and differed by age and year when step-up was made. The most common ICS dose increase was from 200 to 400 µg (budesonide equivalent) which was prescribed in 4,135 children (66% of the ICS dose increase group). Supplementary Table E1 presents the number of children in each of the possible 154 ICS step-up dose categories.

### Changes over time and univariate differences between groups

**Figure 2:**
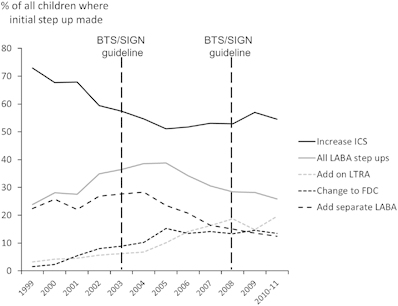
Proportion of step-up options over time in children with asthma already in receipt of inhaled corticosteroid treatment (ICS). All LABA, FDC plus LABA; FDC, fixed dose combination inhaler; LABA, addition of long-acting β_2_-agonist as separate inhaler; LTRA, leukotriene receptor antagonist. Data from 2010 and 2011 are combined since data from the whole of the calendar year 2011 were not available from CPRD. The vertical broken lines indicate when British Thoracic Society/Scottish Intercollegiate Guidelines network (BTS/SIGN) asthma guidelines were published.

The incidence of ICS step-up fell between 1999 and 2005, coinciding with a rise in overall LABA prescribing (that is, FDC plus separate LABA inhaler), but remained static between 2005 and 2011 (Figure 2). The incidence of all LABA prescribing fell and LTRA prescribing rose between 2005 and 2011 (Figure 2 and online Supplementary Table E2). In univariate analyses, the following characteristics at baseline differed across the four treatment groups: age, year when step-up was made, obesity, eczema, daily dose of ICS, medication possession ratio, need for SABA use, oral corticosteroid, and antibiotics in the previous year, primary care consultations, and data source (OPCRD versus CPRD) (Table 3).

### Multivariate analysis

In the multivariate analysis and in comparison with all other groups, children in the FDC group were older and more likely to have seen the GP for asthma (Table 4). The FDC group was more likely to have received oral steroid treatment but less likely to have received antibiotics compared with the LABA and LTRA groups, less likely to be obese and more likely to have regular use of SABA compared with the ICS dose increase group, and more likely to have received an average daily dose of ICS >200  µg (BDP equivalent) than ICS and LTRA groups (Table 4). Children in the LTRA group were more likely to have rhinitis and to have received more than one antibiotic for respiratory symptoms in the previous year (Table 4). Supplementary Table E3 compares the characteristics of children in treatment groups with reference to addition of a separate LABA inhaler.

## Discussion

### Main findings

**Figure 3:**
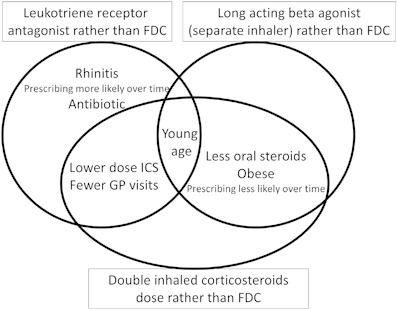
Venn diagram summarising the characteristics of children aged 5–12 with asthma where treatment other than change to fixed dose combination inhaler (FDC) was commenced.

This is the first study to describe which initial ‘step-up’ asthma treatment options are made for 5–12-year-old children in the UK, and also the first to describe the characteristics of children who receive different step-up options. The first main finding was that step-up with increasing ICS dose remained the most popular step-up option between 1999 and 2011 despite national guidelines recommending step-up with LABA before considering increasing ICS dose to 400 µg BUD equivalent per day.^1^ The second novel finding was that children who were stepped up to medication other than LABA had a number of characteristics which were subtly different to those where LABA was introduced (Figure 3), and this suggests that clinicians may be making active decisions not to add LABA. Our results are based on observations from ~15% of children in England and are therefore likely to be generalisable. The hypothesis that children with some characteristics, for example, obesity and rhinitis, gain greater benefit from treatment other than LABA needs to be tested in large populations.

### Strengths and limitations of this study

There are a number of strengths and limitations to this study. Strengths include the large number of individuals included, adjustment for health seeking behaviour, and collection of prescriptions and the use of routine data, which avoids the bias in recruitment which can occur in clinical trials making generalisation unreliable.^14^ A limitation of routinely entered data is that data are incomplete leading to some individuals being excluded. A second limitation to our study is that data were not complete for all individuals studied, for example, BMI was available for 65% of children, and while missing values might not be at random we have demonstrated that children with missing BMI data were equally distributed across step-up groups. A final limitation is that in an observational study such as ours, causation cannot necessarily be inferred from the associations described.

### Interpretation of findings in relation to previously published work

The majority of initial step-up episodes were to increase ICS dose, and over 70% of these were increases to a daily dose of 400 µg budesonide equivalent. Approximately 10% of ICS step-ups were from a daily dose of 100 to 200 µg budesonide, and although these occurred in a younger group of individuals, suggesting that clinicians were being more cautious with ICS dose in younger children, it is possible that some incidents represented ‘fine tuning’ doses from an initially very low value to the dose suggested for step 2 treatment.^1^ Notwithstanding this small number of step-ups to 200 µg, the majority of ICS step-ups were consistent with a move to the BTS/SIGN step 3 treatment step but these episodes were not preceded with LABA treatment and this is not consistent with the guideline.^1^

Table 2 demonstrates that the majority (69%) of ICS step-up options were from 200 to 400 µg budesonide per day, and this suggests that that clinicians looking after patients in the real world have found that stepping up from low to intermediate ICS dose is a safe and effective option and that this accounts for its relative popularity. Eight percent of ICS step-ups were either from low direct to high dose or to ICS dose in excess of the ceiling dose of 800 µg budesonide equivalent and these observations replicate our earlier work highlighting the prevalence of high dose ICS prescribing.^2^ The BTS/SIGN^1^ guideline suggests that children in receipt of high dose ICS (that is, 800 µg budesonide per day) should receive specific written advice about steroid replacement and be under the care of a specialist paediatrician.

Asthma prescribing is known to deviate from guidelines in the UK,^21,22^ and other countries,^23,24^ and might in part be due to a delay between guidelines being published and implemented; other reasons for prescribing changes include falls in pricing as medications come off patent and concerns about adverse effects of LABA therapy raised by the United States Food and Drug Administration in 2005.^25^ The BTS/SIGN guidelines first recommend LABA addition rather than increasing ICS dose since 1995,^26^ and this change in advice might explain the continuing rise in LABA prescribing between 1999 and 2005. The 2003 BTS/SIGN guideline was the first to recommend inclusion of LTRA^27^ but this advice was anticipated since 100,000 LTRA prescriptions for children were made in 2003^22^ and we see rising LTRA prescribing in the early 2000s. What is notable is that despite LABA being advocated as the first step by guidelines since 1995, the rise in LABA prescribing pre 2006 has faltered, as has been demonstrated previously^2,22^ and here we show a decline in LABA prescribing between 2006 and 2011; this study cannot explain why LABAs are less frequently prescribed but one explanation might be that prescribers find LTRA or increasing ICS dose to be more effective. An alternative explanation for increasing step-up by addition of LTRA or increasing ICS in younger children within the 5–12-year-old category may be ‘carry over’ of more recent BTS/SIGN^1^ and GINA^12^ guideline advice to use these step-up options in the <5-year-old category.

### Implications for future research, policy and practice

The presence of differences between individuals in the step-up option groups suggests that prescribers perceive that some individuals gain benefit from specific step-up options. In a tertiary care setting, clinicians have previously been shown to prescribe asthma medications by phenotype, that is, viral wheeze versus multitrigger wheeze,^28^ with the implication that some individuals are recognised as having preferential response to some asthma medications. In one study, differential response to step-up therapy has been linked to age, the presence of eczema and ethnicity,^5^ while in a second study increased reliever medication use, increased exhaled nitric oxide, and reduced lung function were more likely following increasing ICS dose compared with the addition of LTRA.^10^ Obese children are less responsive to ICS therapy,^29^ but obese children were 40% more likely to have ICS step-up rather than addition of FDC in our study. We observed that children stepped up to LTRA had a higher prevalence of rhinitis and a 70% increased risk for multiple prescriptions for antibiotics for respiratory symptoms, when compared with both LABA step-up options, and this decision may be justifiable since LTRA are known to be effective against upper and lower respiratory tract symptoms.^30^ Our study raises at least two research questions which we will address in future work: (i) what are the outcomes of stepping up asthma treatment using FDC as the gold standard in comparison with increasing ICS or addition of LTRA or LABA separately?; (ii) are there patient subgroups (or phenotypes) who gain preferential response from options other than step-up to FDC?

### Conclusions

Guidelines are one of the critical components of good clinical practice but are limited by their one-size-fits-all approach and, in the case of stepping up asthma treatment in children, often lack an evidence base. The clinical setting of a child whose asthma symptoms are not adequately controlled with ICS is common and there is a lack of evidence for ‘best practice’; in this context it is appropriate for clinicians to consider guideline advice but not necessarily apply this advice. Our results demonstrate that clinicians in the UK frequently do not adhere to the BTS/SIGN guideline for asthma,^1^ and deviation from recommendations appears to be an active decision in circumstances where a number of coexisting factors are present. An individualised approach may be more effective in guiding the treatment of asthma beyond step 2 (ICS therapy), which is based around an asthma action plan and in future guided by genetic factors^31^ or biomarkers such as exhaled nitric oxide.^32^

## Figures and Tables

**Table 1:** Characteristics of the 10,793 children with asthma in receipt of inhaled steroid treatment where a first step-up was made

	n* (%)*
Median age (Interquartile range), years	9 (6, 11)
Female gender	4,457 (41.3%)
	
*BMI centile*	
<91th	4,241 (39.3%)
91–97th	825 (7.6%)
⩾98th	869 (8.1%)
Missing	4,858 (45.0%)
Rhinitis diagnosis	2,497 (23.1%)
Eczema drugs	5,360 (49.7%)
Asthma diagnosis	10,447 (96.8%)
Median year of index date (Interquartile range)	2004 (2002, 2007)
	
*Time of first asthma prescription*	
Years before IPD:	
0–1	3,460 (32.1%)
2–3	3,056 (28.3%)
4–5	2,314 (21.4%)
6+	1,963 (18.2%)
	
*Average ICS daily dosage * *(µg)* a	
>0–100	5,028 (46.6)
101–200	3,392 (31.4)
201+	2,373 (22.0)
Medication possession ratio ⩾80%^a^	2,479 (23.0%)
Any SABA prescription^a^	10,294 (95.4%)
	
*Mean dail* *y SABA dosage (µg)* a	
0	499 (4.6%)
>0–200	5,874 (54.4%)
201+	4,420 (41.0%)
Acute oral steroid prescription^a^	1,034 (9.6%)
Asthma-related out-patient visit^a^	99 (0.9%)
Asthma-related in-patient visit^a^	51 (0.5%)
Asthma-related A&E visit^a^	56 (0.5%)
	
*Antibiotics with evidence of respiratory review* a	
0	7,656 (70.9%)
1	2,107 (19.5%)
2+	1,030 (9.5%)
	
*GP consultations* *for asthma* ^a^	
0	2,655 (24.6%)
1	3,113 (28.8%)
2	2,419 (22.4%)
3+	2,606 (24.2%)
	
*GP consultations not for asthma* ^a^	
0	1,117 (10.4%)
1–2	3,033 (28.1%)
3–5	3,553 (32.9%)
6+	3,090 (28.6%)

The table displays number of children (*n*) unless specified otherwise.

Abbreviations: A&E=accident and emergency; GP=general practitioner; ICS, inhaled corticosteroids; IPD=index prescribing date; SABA, short-acting β_2_-agonist;.

a These variables are with respect to the 12 months prior to step-up.

**Table 2:** Characteristics of children where ICS dose was stepped up by different increments

*Dose prior to step-up to dose after * *step-up, µg* * BDP equivalent (*n*)*	*Mean (s.d.) index year*	*Male, *n* (%)*	*Mean (s.d.) age, years*	*Rhinitis diagnosis, *n* (%)*	*Eczema drugs, *n* (%)*	*SABA prescription, *n* (%)*	*Median (IQR) daily SA* *BA dosage, µg*	*Acute oral steroid prescription, *n* (%)*	*Antibiotics prescription*^a^*, *n* (%)*
100–200 (732)	2004.7 (3.4)	421 (57.5)	8.0 (2.1)	143 (19.5)	386 (52.7)	684 (93.4)	147.9 (54.8, 219.2)	64 (8.7)	206 (28.1)
100–400 (265)	2003.8 (3.1)	158 (59.6)	8.3 (2.3)	53 (20.0)	138 (52.1)	250 (94.3)	164.4 (109.6, 274.0)	21 (7.9)	82 (30.9)
200–400 (4,135)	2004.4 (3.3)	2,418 (58.5)	8.5 (2.3)	959 (23.2)	2,091 (50.6)	3,896 (94.2)	164.4 (54.8, 274.0)	337 (8.1)	1,148 (27.8)
200–800 (204)	2003.0 (3.3)	120 (58.8)	8.4 (2.2)	45 (22.1)	94 (46.1)	184 (90.2)	109.6 (54.8, 274.0)	15 (7.4)	45 (22.1)
200–1,000 (80)	2003.1 (3.3)	40 (50.0)	7.4 (2.2)	16 (20.0)	42 (52.5)	77 (96.3)	219.2 (109.6, 328.8)	5 (6.3)	16 (20.0)
400–800 (364)	2003.5 (3.3)	215 (59.1)	9.2 (2.3)	75 (20.6)	163 (44.8)	346 (95.1)	147.9 (104.1, 274.0)	30 (8.2)	104 (28.6)
400–1000 (194)	2002.5 (2.7)	120 (61.9)	8.5 (2.5)	47 (24.2)	83 (50.2)	184 (94.8)	164.4 (54.8, 274.0)	18 (9.3)	49 (25.3)
*P *value^b^	<0.001	0.71	<0.001	0.31	0.05	0.26	0.27	0.98	0.25

The seven increments described account for 96% of all ICS step-ups.

Abbreviations: BDP, budesonide diproprionate; ICS, inhaled corticosteroids; IQR, interquartile range; SABA, short-acting β_2_-agonist.

a Antibiotics prescribed with evidence of respiratory review.

b *Χ*^2^-test for categorical variables and Kruskal–Wallis for variables measured on the interval or ratio scale.

**Table 3:** Patient characteristics according to first step-up therapy during 1999–2011

	*FDC (*n*=1,107)*	*Add LABA (*n*=2,329)*	*Increase ICS dose (*n*=6,252)*	*Add LTRA (*n*=1,105)*	P *value*^a^
Median (IQR) age, years	10 (8, 11)	9 (6, 11)	8 (6, 10)	8 (6, 10)	<0.001
Male sex	660 (59.6)	1,343 (57.7)	3,663 (58.6)	670 (60.6)	0.37
					
*Median (IQR) BMI centile*					
Median (IQR)	65 (29, 93)	68 (28, 94)	67 (31, 93)	65 (31, 93)	0.13
Missing, *n* (%)	457 (41.3)	1,076 (46.2)	2,875 (46.0)	450 (40.7)	
					
*BMI centile, categorised*					
<91th	467 (42.2)	881 (37.8)	2427 (38.8)	466 (42.2)	<0.001
91–97th	102 (9.2)	175 (7.5)	440 (7.0)	108 (9.8)	
⩾98th	81 (7.3)	197 (8.5)	510 (8.2)	81 (7.3)	
Missing	457 (41.3)	1,076 (46.2)	2,875 (46.0)	450 (40.7)	
Rhinitis diagnosis	243 (22.0)	575 (24.7)	1,404 (22.5)	275 (24.9)	0.06
Eczema drugs	552 (49.9)	1,085 (46.6)	3,149 (50.4)	574 (51.9)	0.006
Paracetamol prescription	162 (14.6)	316 (13.6)	915 (14.6)	175 (15.8)	0.34
Asthma diagnosis	1,073 (96.9)	2,247 (96.5)	6,067 (97.0)	1,060 (95.9)	0.19
Median (IQR) index year	2006 (2004, 2008)	2004 (2002, 2006)	2004 (2001, 2007)	2007 (2004, 2008)	<0.001
First asthma prescription >1 year before index date	771 (69.7)	1,552 (66.6)	4,258 (68.1)	752 (68.1)	0.34
					
*Average ICS daily dosag* *e (µg)*					
>0–100	379 (34.2)	792 (34.0)	3,400 (54.4)	457 (41.4)	<0.001
101–200	387 (35.0)	818 (35.1)	1,811 (29.0)	376 (34.0)	
201+	341 (30.8)	719 (30.9)	1,041 (16.7)	272 (24.6)	
					
*Medication possession ratio*					
⩾80%	211 (19.1)	506 (21.7)	1,516 (24.2)	246 (22.3)	<0.001
					
*SABA prescription*					
1+	1,083 (97.8)	2,261 (97.1)	5,883 (94.1)	1,067 (96.6)	<0.001
					
*Mean daily SABA dosa* *ge (µg)*					
0	24 (2.2)	68 (2.9)	369 (5.9)	38 (3.4)	<0.001
>0–200	584 (52.8)	1,229 (52.8)	3,478 (55.6)	583 (52.8)	
201+	499 (45.1)	1,032 (44.3)	2,405 (38.5)	484 (43.8)	
					
*Acute oral steroid use*					
⩾1	156 (14.1)	237 (10.2)	512 (8.2)	129 (11.7)	<0.001
					
*Asthma-related out-patient visit*					
⩾1	14 (1.3)	18 (0.8)	48 (0.8)	19 (1.7)	0.01
					
*Asthma-related In-patient visit*					
⩾1	11 (1.0)	9 (0.4)	22 (0.4)	9 (0.8)	0.01
					
*Asthma-related A&E visit*					
⩾1	6 (0.5)	6 (0.3)	36 (0.6)	8 (0.7)	
					
*Antibiotics with evidence of respiratory review*					
0	800 (72.3)	1,631 (70.0)	4,523 (72.3)	702 (63.5)	<0.001
1	210 (19.0)	470 (20.2)	1,199 (19.2)	228 (20.6)	
2+	97 (8.8)	228 (9.8)	530 (8.5)	175 (15.8)	
					
*GP consultations for asthma*					
0	180 (16.3)	524 (22.5)	1,663 (26.6)	288 (24.6)	<0.001
1	297 (26.8)	580 (24.9)	1,950 (31.2)	286 (25.9)	
2	249 (22.5)	530 (22.8)	1,405 (22.5)	235 (21.3)	
3+	381 (34.4)	695 (29.8)	1,234 (19.7)	296 (26.8)	
					
*GP consultations not for asthma*					
0	99 (8.9)	240 (10.3)	713 (11.4)	65 (5.9)	<0.001
1–2	314 (28.4)	618 (26.5)	1,847 (29.5)	254 (23.0)	
3–5	359 (32.4)	800 (34.3)	2,033 (32.5)	361 (32.7)	
6+	335 (30.3)	671 (28.8)	1,659 (26.5)	425 (38.5)	
OPCRD data source (versus CPRD)	740 (66.8)	1,709 (73.4)	4,378 (70.0)	782 (70.8)	<0.001

Numbers denote *n* (%) unless specified otherwise.

Abbreviations: BMI, body mass index; CPRD, Clinical Practice Research Datalink; FDC, fixed dose combination inhaler; ICS, inhaled corticosteroids; IQR, interquartile range; LABA, long-acting β_2_-agonist; LTRA, leukotriene receptor antagonist; OPCRD, Optimum Patient Care Research Database.

a *Χ* ^2^-test for categorical variables and Kruskal–Wallis for variables measured on the interval or ratio scale.

**Table 4:** Multivariate associations between patient characteristics and step-up treatment in children with asthma whose treatment was stepped up from ICS treatment with reference to change to fixed dose combination inhaler

	*Add LABA (*n*=2,329)*		*Increase ICS dose (*n*=6,252)*		*Add LTRA (*n*=1,105)*		P *value*
	*OR*	*95% CI*	*OR*	*95% CI*	*OR*	*95% CI*	

Age per year	0.87	0.84, 0.90	0.86	0.83, 0.88	0.79	0.76, 0.82	<0.001
							
*BMI centile*							
<91th	1.00		1.00		1.00		0.05
91–97th	1.02	0.78, 1.35	0.96	0.75, 1.23	1.24	0.91, 1.68	
⩾98th	1.45	1.08, 1.93	1.41	1.08, 1.83	1.14	0.81, 1.60	
missing	1.06	0.90, 1.25	0.98	0.85, 1.14	1.00	0.83, 1.21	
							

Index year per year	0.83	0.81, 0.85	0.85	0.83, 0.87	1.05	1.02, 1.08	<0.001
Rhinitis diagnosis	1.10	0.92, 1.31	1.02	0.86, 1.19	1.30	1.06, 1.59	0.01
Eczema drugs	0.89	0.77, 1.04	1.12	0.98, 1.28	1.00	0.84, 1.18	<0.001
							
*Average ICS daily dosag* *e (µg)*							
>0–100	1.00		1.00		1.00		<0.001
101–200	1.00	0.83, 1.20	0.50	0.43, 0.59	0.83	0.68, 1.03	
201+	0.90	0.73, 1.10	0.29	0.24, 0.35	0.69	0.54, 0.87	
							
*SABA daily dosag* *e (µg)*							
0	1.00		1.00		1.00		<0.001
>0–200	0.90	0.56, 1.47	0.40	0.26, 0.62	0.63	0.37, 1.07	
201+	0.98	0.60, 1.59	0.55	0.36, 0.86	0.73	0.42, 1.24	
							
*Acute oral steroid use*							
1+	0.71	0.56, 0.90	0.68	0.55, 0.84	0.89	0.68, 1.17	0.001
							
*Asthma-related out-patient visit*							
1+	0.59	0.29, 1.22	0.58	0.31, 1.08	1.14	0.56, 2.33	0.06
							
*Antibiotics with evidence of respiratory review*							
0	1.00		1.00		1.00		0.008
1	1.21	1.00, 1.47	1.15	0.96, 1.37	1.14	0.91, 1.43	
2+	1.27	0.96, 1.67	1.21	0.94, 1.56	1.68	1.25, 2.25	
							
*GP consultations* *for asthma*							
0	1.00		1.00		1.00		<0.001
1	0.86	0.69, 1.08	0.87	0.71, 1.06	0.61	0.47, 0.78	
2	0.97	0.76, 1.22	0.78	0.63, 0.97	0.59	0.45, 0.77	
3+	0.74	0.59, 0.93	0.45	0.37, 0.55	0.46	0.36, 0.60	
							
*GP consultations not for asthma*							
0	1.00		1.00		1.00		0.01
1–2	0.91	0.69, 1.21	0.89	0.69, 1.14	1.20	0.84, 1.72	
3–5	1.12	0.85, 1.47	0.91	0.71, 1.17	1.32	0.93, 1.88	
6+	1.04	0.78, 1.38	0.84	0.64, 1.08	1.35	0.94, 1.94	

Abbreviations: BMI, body mass index; CI, confidence interval; ICS, inhaled corticosteroids; LABA, long-acting β_2_-agonist; LTRA, leukotriene receptor antagonist; OR, odds ratio; SABA, short-acting β_2_-agonist.

## Notes

MT: Neither MT nor any member of his close family has any shares in pharmaceutical companies. In the last 3 years he has received speaker’s honoraria for speaking at sponsored meetings or satellite symposia at conferences from the following companies marketing respiratory and allergy products: Aerocrine, Astra Zeneca, Boehringer Inglehiem, GSK, MSD, and Teva. He has received honoraria for attending advisory panels with; Aerocrine, Almirall, Astra Zeneca, BI, Chiesi, GSK, MSD, and Novartis. He has received sponsorship to attend international scientific meetings from: GSK, Astra Zeneca, and Mundipharma. He has received funding for research projects from: GSK and Almirall. He is a chief medical adviser to the charity Asthma UK, a member of the BTS SIGN Asthma guideline group and the NICE Asthma guideline group. DP: board Membership: Aerocrine, Almirall, Amgen, AstraZeneca, Boehringer Ingelheim, Chiesi, Meda, Mundipharma, Napp, Novartis, and Teva. Consultancy: Almirall, Amgen, AstraZeneca, Boehringer Ingelheim, Chiesi, GlaxoSmithKline, Meda, Mundipharma, Napp, Novartis, Pfizer, and Teva. Grants/Grants Pending: UK National Health Service, British Lung Foundation, Aerocrine, AstraZeneca, Boehringer Ingelheim, Chiesi, Eli Lilly, GlaxoSmithKline, Meda, Merck, Mundipharma, Novartis, Orion, Pfizer, Respiratory Effectiveness Group, Takeda, Teva, and Zentiva. Payments for lectures/speaking: Almirall, AstraZeneca, Boehringer Ingelheim, Chiesi, Cipla, GlaxoSmithKline, Kyorin, Meda, Merck, Mundipharma, Novartis, Pfizer, SkyePharma, Takeda, and Teva. Payment for manuscript preparation: Mundipharma and Teva. Patents (planned, pending or issued): AKL. Payment for the development of educational materials: GSK and Novartis. Stock/Stock options: Shares in AKL which produces phytopharmaceuticals and owns 80% of Research in Real Life and its subsidiary social enterprise Optimum Patient Care. Payment for travel/accommodations/meeting expenses: Aerocrine, Boehringer Ingelheim, Mundipharma, Napp, Novartis, and Teva. Funding for patient enrolment or completion of research: Almirall, Chiesi, Teva, and Zentiva. Peer reviewer for grant committees: Medical Research Council (2014), Efficacy and Mechanism Evaluation programme (2012), and HTA (2014). Unrestricted funding for investigator-initiated studies: Aerocrine, AKL, Almirall, Boehringer Ingelheim, Chiesi, Meda, Mundipharma, Napp, Novartis, Orion, Takeda, Teva, and Zentiva. AB is an employee of RiRL, which has conducted paid research in respiratory disease on behalf of the following organisations in the past 5 years: Aerocrine, AKL Ltd, Almirall, Boehringer Ingelheim, Chiesi, GlaxoSmithKline, Meda, Mundipharma, Napp, Novartis, Orion, Takeda, Teva, and Zentiva. ST, CM, and KR have no conflicts of interest to declare.
