# Supplementary material for: Initial step-up treatment changes in asthmatic children already prescribed inhaled corticosteroids: a historical cohort study
Source: NPJ Prim Care Respir Med. 2015 Jun 11;25:15041–. doi: 10.1038/npjpcrm.2015.41 (PMC4498242; doi:10.1038/npjpcrm.2015.41)
Supplement: Supplementary Information [file npjpcrm201541-s1.doc]

Step up treatment options in children with asthma in receipt of inhaled corticosteroids – which option to which child?

On line supplement

**METHODS**

**Definitions used**

The average daily ICS dosage is the sum of inhalers multiplied by doses in pack and strength (microg budesonide diproprionate [BDP] equivalent) divided by 365. Average daily short acting beta agonist (SABA) use during the year was calculated as the average number of puffs per day over the year multiplied by dose (in mcg) for the inhaler used. SABA use was defined as any prescription in the baseline year. Acute oral steroid use was defined as courses that were not maintenance therapy (daily dosing instructions of <10mg Prednisolone or prescriptions for 1mg Prednisolone tablets), and/or where dosing instructions suggest exacerbation treatment (e.g. 6,5,4,3,2,1 reducing, or 30mg as directed), and/or with no dosing instructions, but unlikely to be maintenance therapy with a code for asthma or a lower respiratory event. Medication possession ratio was calculated as the number of days supply of drug divided by 365 multiplied by 100 and expressed as <80% (non adherent) and ≥80% (adherent) [1]. Body mass index was derived from height and weight and converted to centiles and z scores with reference to the 1990 UK standard [2]. In accordance with previous work [3] values exceeding +5 and -5 z scores were excluded as presumed mistake in data entry and clinical cut offs for overweight (≥91^st^ centile) and obese (≥98^th^ centile) were applied.

|  | **ICS dose after step up (BDP equivalent dose)** | | | | | | | | | | | | | | |
| --- | --- | --- | --- | --- | --- | --- | --- | --- | --- | --- | --- | --- | --- | --- | --- |
| **Dose before step up (BDP equivalent dose)** | **200** | **300** | **400** | **500** | **600** | **750** | **800** | **900** | **1000** | **1200** | **1500** | **1600** | **1800** | **2000** | **Total** |
| **50** | 10 | 0 | 5 | 0 | 0 | 0 | 0 | 0 | 0 | 0 | 0 | 0 | 0 | 0 | 15 |
| **100** | 732 | 2 | 265 | 5 | 3 | 0 | 12 | 0 | 14 | 0 | 0 | 1 | 0 | 0 | 1034 |
| **150** | 0 | 0 | 2 | 0 | 0 | 0 | 0 | 0 | 0 | 0 | 0 | 0 | 0 | 0 | 2 |
| **200** | 0 | 29 | 4135 | 38 | 21 | 0 | 204 | 0 | 80 | 3 | 1 | 14 | 0 | 6 | 4531 |
| **250** | 0 | 0 | 3 | 1 | 0 | 0 | 0 | 0 | 1 | 0 | 0 | 0 | 0 | 0 | 5 |
| **300** | 0 | 0 | 0 | 0 | 3 | 0 | 2 | 0 | 0 | 0 | 0 | 0 | 0 | 0 | 5 |
| **400** | 0 | 0 | 0 | 0 | 19 | 2 | 364 | 1 | 194 | 2 | 1 | 7 | 1 | 19 | 610 |
| **500** | 0 | 0 | 0 | 0 | 0 | 0 | 3 | 0 | 24 | 0 | 0 | 0 | 0 | 1 | 28 |
| **600** | 0 | 0 | 0 | 0 | 0 | 0 | 0 | 0 | 1 | 0 | 0 | 0 | 0 | 0 | 1 |
| **800** | 0 | 0 | 0 | 0 | 0 | 0 | 0 | 0 | 0 | 3 | 0 | 2 | 0 | 5 | 10 |
| **1000** | 0 | 0 | 0 | 0 | 0 | 0 | 0 | 0 | 0 | 0 | 0 | 0 | 0 | 11 | 11 |
| **Total** | 742 | 31 | 4410 | 44 | 46 | 2 | 585 | 1 | 314 | 8 | 2 | 24 | 1 | 42 | 6252 |

Table E1. Number of children with asthma in receipt of inhaled corticosteroid (ICS) treatment where ICS was increased with stratification by ICS dose before and after ICS step up.

Table E2. Number of children in each step up group for each year between 1999-2011.

|  | | TOTAL | By Treatment Group, n(%) | | | |
| --- | --- | --- | --- | --- | --- | --- |
|  |  |  | Increase  in ICS | Change  TO FDC | Add on  OF LABA | Add on  OF LTRA |
| Year of IPD | 1999 | 747 | 545 (73.0) | 11 (1.5) | 167 (22.4) | 24 (3.2) |
|  | 2000 | 767 | 519 (67.7) | 18 (2.3) | 198 (25.8) | 32 (4.2) |
|  | 2001 | 942 | 640 (67.9) | 51 (5.4) | 208 (22.1) | 43 (4.6) |
|  | 2002 | 994 | 590 (59.4) | 80 (8.0) | 267 (26.9) | 57 (5.7) |
|  | 2003 | 923 | 529 (57.3) | 82 (8.9) | 255 (27.6) | 57 (6.2) |
|  | 2004 | 1063 | 580 (54.6) | 110 (10.3) | 301 (28.3) | 72 (6.8) |
|  | 2005 | 1044 | 534 (51.1) | 160 (15.3) | 245 (23.5) | 105 (10.1) |
|  | 2006 | 973 | 504 (51.8) | 131 (13.5) | 201 (20.7) | 137 (14.1) |
|  | 2007 | 1010 | 536 (53.1) | 143 (14.2) | 166 (16.4) | 165 (16.3) |
|  | 2008 | 875 | 463 (52.9) | 117 (13.4) | 131 (15.0) | 164 (18.7) |
|  | 2009 | 756 | 431 (57.0) | 110 (14.6) | 103 (13.6) | 112 (14.8) |
|  | 2010 | 560 | 327 (58.4) | 62 (11.1) | 63 (11.3) | 108 (19.3) |
|  | 2011 | 139 | 54 (38.8) | 32 (23.0) | 24 (17.3) | 29 (20.9) |
|  | Total | 10793 | 6252 (57.9) | 1107 (10.3) | 2329 (21.6) | 1105 (10.2) |

Table E3. Multivariate associations between patient characteristics and step up treatment in children with asthma whose treatment was stepped up from inhaled corticosteroid (ICS) treatment with reference to change to long acting beta agonist as separate inhaler. FDC= Fixed Dose Combination inhaler, LTRA=Leukotriene Receptor Antagonist.

.

|  |  | Change to FDC (n=1119) | | Increase ICS dose (n=6414) | | Add LTRA (n=1107) | | p-value |
| --- | --- | --- | --- | --- | --- | --- | --- | --- |
|  |  | OR | 95% CI | OR | 95% CI | OR | 95% CI |  |
| Age | per year | 1.16 | 1.12, 1.20 | 0.99 | 0.97, 1.01 | 0.91 | 0.88, 0.94 | <.001 |
| BMI | < 91th | 1.00 |  | 1.00 |  | 1.00 |  | 0.06 |
|  | 91-97th | 0.98 | 0.74, 1.29 | 0.94 | 0.77, 1.15 | 1.22 | 0.93, 1.60 |  |
|  | ≥ 98th | 0.69 | 0.52, 0.92 | 0.97 | 0.81, 1.18 | 0.79 | 0.59, 1.05 |  |
|  | missing | 0.95 | 0.80, 1.11 | 0.93 | 0.83, 1.04 | 0.95 | 0.80, 1.12 |  |
| Index year | per year | 1.21 | 1.18, 1.24 | 1.02 | 1.01, 1.04 | 1.26 | 1.23, 1.29 | <.001 |
| Rhinitis diagnosis |  | 0.91 | 0.76, 1.09 | 0.92 | 0.82, 1.04 | 1.18 | 0.99, 1.40 | 0.01 |
| Eczema drugs |  | 1.12 | 0.97, 1.30 | 1.25 | 1.13, 1.38 | 1.12 | 0.96, 1.30 | <.001 |
| Average ICS daily dosage (mcg) | >0-100 | 1.00 |  | 1.00 |  | 1.00 |  | <.001 |
|  | 101-200 | 1.00 | 0.84, 1.20 | 0.50 | 0.45, 0.56 | 0.84 | 0.70, 1.00 |  |
|  | 201+ | 1.11 | 0.91, 1.36 | 0.32 | 0.28, 0.37 | 0.76 | 0.62, 0.94 |  |
| SABA daily dosage (mcg) | 0 | 1.00 |  | 1.00 |  | 1.00 |  | <.001 |
|  | >0-200 | 1.11 | 0.68, 1.79 | 0.45 | 0.34, 0.59 | 0.69 | 0.45, 1.06 |  |
|  | 201+ | 1.02 | 0.63, 1.66 | 0.57 | 0.43, 0.75 | 0.74 | 0.48, 1.13 |  |
| Acute Oral Steroid use | 1+ | 1.41 | 1.12, 1.78 | 0.96 | 0.80, 1.14 | 1.26 | 0.98, 1.61 | <.001 |
| Asthma related Out Patient visit | 1+ | 1.69 | 0.82, 3.47 | 0.98 | 0.56, 1.70 | 1.93 | 0.99, 3.76 | 0.06 |
| Antibiotics with evidence of  respiratory review | 0 | 1.00 |  | 1.00 |  | 1.00 |  | 0.009 |
|  | 1 | 0.83 | 0.68, 1.00 | 0.95 | 0.83, 1.08 | 0.94 | 0.78, 1.14 |  |
|  | 2+ | 0.79 | 0.60, 1.04 | 0.96 | 0.80, 1.15 | 1.33 | 1.04, 1.69 |  |
| GP Consultations for asthma | 0 | 1.00 |  | 1.00 |  | 1.00 |  | <.001 |
|  | 1 | 1.16 | 0.93, 1.46 | 1.01 | 0.88, 1.16 | 0.71 | 0.57, 0.87 |  |
|  | 2 | 1.03 | 0.82, 1.31 | 0.81 | 0.70, 0.94 | 0.61 | 0.49, 0.76 |  |
|  | 3+ | 1.35 | 1.08, 1.70 | 0.61 | 0.53, 0.70 | 0.62 | 0.50, 0.77 |  |
| GP consultations not for asthma | 0 | 1.00 |  | 1.00 |  | 1.00 |  | 0.01 |
|  | 1-2 | 1.10 | 0.83, 1.45 | 0.97 | 0.81, 1.16 | 1.31 | 0.95, 1.80 |  |
|  | 3-5 | 0.89 | 0.68, 1.18 | 0.81 | 0.68, 0.97 | 1.18 | 0.86, 1.62 |  |
|  | 6+ | 0.97 | 0.72, 1.29 | 0.81 | 0.67, 0.97 | 1.30 | 0.95, 1.80 |  |

**REFERENCES**

1 Brooks CM, Richards JM, Kohler CL*, et al*. Assessing adherence to asthma medication and inhaler regimens: a psychometric analysis of adult self-report scales. *Med Care.* 1994;**32**:298-307.

2 Cole TJ, Freeman JV, Preece MA. Body mass index reference curves for the UK, 1990. *Arch Dis Child.* 1995;**73**:25-29.

3 Smith SM, Craig LC, Raja AE*, et al*. Growing up before growing out: secular trends in height, weight and obesity in 5--6-year-old children born between 1970 and 2006. *Arch Dis Child.* 2013;**98**:269-273.
